# Stain-free enucleation of mouse and human oocytes with a 1033 nm femtosecond laser

**DOI:** 10.1117/1.JBO.29.6.065002

**Published:** 2024-05-29

**Authors:** Alina A. Osychenko, Alexandr D. Zalessky, Alexey V. Bachurin, David Yu. Martirosyan, Maria S. Egorova, Viktor A. Nadtochenko

**Affiliations:** a N.N. Semenov Federal Research Center for Chemical Physics Russian Academy of Sciences, Moscow, Russia; b Medical Center of ART, Moscow, Russia

**Keywords:** recipient cytoplast, enucleation, femtosecond laser, spindle

## Abstract

**Significance:**

Preparation of a recipient cytoplast by oocyte enucleation is an essential task for animal cloning and assisted reproductive technologies in humans. The femtosecond laser is a precise and low-invasive tool for oocyte enucleation, and it should be an appropriate alternative to traditional enucleation by a microneedle aspiration. However, until recently, the laser enucleation was performed only with applying a fluorescent dye.

**Aim:**

This work is aimed to (1) achieve femtosecond laser oocyte enucleation without applying a fluorescent dye and (2) to study the effect of laser destruction of chromosomes on the structure and dynamics of the spindle.

**Approach:**

We applied polarized light microscopy for spindle visualization and performed stain-free mouse and human oocyte enucleation with a 1033 nm femtosecond laser. Also, we studied transformation of a spindle after metaphase plate elimination by a confocal microscopy.

**Results:**

We demonstrated a fundamental possibility of inactivating the metaphase plate in mouse and human oocytes by 1033 nm femtosecond laser radiation without applying a fluorescent dye. Irradiation of the spindle area, visualized by polarized light microscopy, resulted in partly or complete metaphase plate destruction but avoided the microtubules impairment. After the metaphase plate elimination, the spindle reorganized, however, it was not a complete depolymerization.

**Conclusions:**

This method of recipient cytoplast preparation is expected to be useful for animal cloning and assisted reproductive technologies.

## 1 Introduction

Inactivation of the oocyte metaphase plate using a near-infrared picosecond or femtosecond laser is an efficient and accurate method to prepare recipient cytoplasts.^1^^–^^3^ A recipient cytoplast (an oocyte without chromosomes) is required for animal cloning and assisted reproductive technologies in humans, viz., for mitochondrial replacement therapy.^4^^,^^5^ Removal of chromosomes lined up on the metaphase plate of the oocyte is commonly called enucleation.^6^

**Fig. 1:**
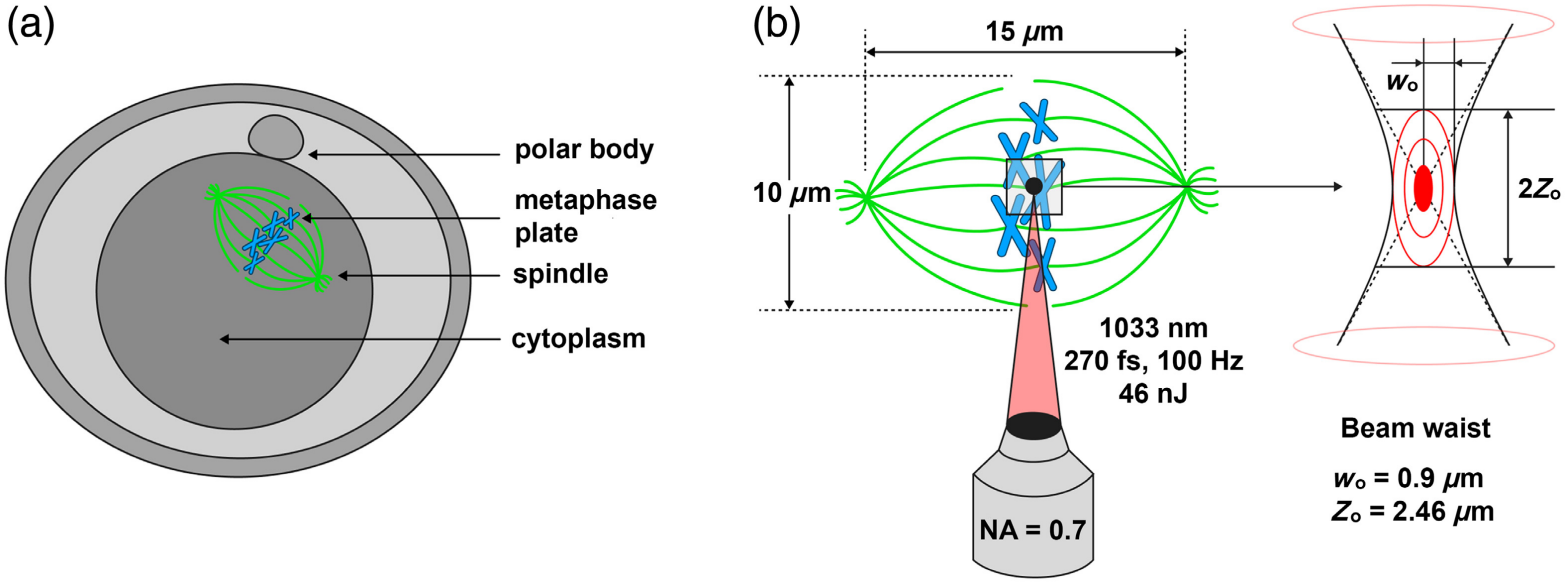
(a) Typical structure of metaphase II oocytes. (b) The scheme of the laser action on the metaphase plate. The laser beam is focused on the chromosomes of a metaphase plate (right), and the laser absorption occurs within the beam waist. The beam waist radius $w_{0}$ is 0.9  μm, and Rayleigh distance $Z_{0}$ is 2.46  μm.

Oocytes in metaphase II of meiosis have homogeneous cytoplasm, and the metaphase plate is invisible in bright field and differential interference contrast (DIC). Metaphase II oocytes have typical structure, as shown in Fig. 1(a). In traditional enucleation, the metaphase plate is localized by a polar body^7^ or as a translucent region,^8^^,^^9^ and then aspirated with a minimal volume of cytoplasm. The spindle as well as reprogramming factors, e.g., cyclin B1 and p34^cdc2^,^10^ ORF1,^11^ and soronin,^12^ are also aspirated from the cell.

Laser enucleation of oocytes appears less harmful compared to the traditional microneedle method of metaphase plate aspiration, since it does not require the cell puncture and avoids the loss of reprogramming factors. The scheme of the laser action on the metaphase plate is given in Fig. 1(b). Using a tightly focused near-infrared femtosecond laser beam, DNA can be eliminated in a living oocyte without significantly affecting cell viability.^13^ This becomes possible due to non-linear nature of femtosecond laser absorption by biological material as absorption occurs only in the region of highest laser intensity (laser focal spot). This provides a possibility of highly localized treatment. During nonlinear adsorption of femtosecond laser action, highly localized formation of low-density plasma occurs.^14^ Interaction between low-density plasma and biological material leads to desirable localized DNA destruction.^15^

Laser enucleation requires accurate detection of the metaphase plate. Typically, oocytes are stained with vital DNA-specific dye Hoechst, which has excitation/emission maxima of 350/461 nm and are subjected to UV illumination for visualization.^2^^,^^3^^,^^13^ In terms of human oocyte enucleation, laser destruction of DNA without dye (hereinafter, stain-free) is of special interest. Enucleation of oocytes using polarized microscopy has already been performed in mice^16^ and humans,^17^ although this was aspiration with an injection pipette. In this work, we applied polarized light microscopy to detect the spindle and performed stain-free laser enucleation of mouse and human oocytes.

A key advantage of laser enucleation over aspiration is the ability to retain all intracellular compartments surrounding the metaphase plate, i.e., the spindle and reprogramming factors. However, when the metaphase plate is irradiated, the spindle can also be impaired because microtubules tightly surround the chromosomes at this stage of oocyte development. Moreover, it is known that the centromeres of each chromosome together with a kinetochore act as microtubule-organizing centers (MTOCs).^18^ Spindle integrity and proper kinetochore-microtubule attachment are essential for successful metaphase-to-anaphase transition (spindle checkpoint).^19^ Therefore, it is important to study spindle transformation after laser destruction of chromosomes. This work also studied the effect of laser destruction of chromosomes on the structure and dynamics of the spindle.

## 2 Materials and Methods

### 2.1 Oocyte Collection and Preparation

#### 2.1.1 Mouse oocyte collection

Animal and oocyte preparation was performed according to a standard scheme described in our previous work.^13^ 6 to 8 week old C57BL6/CBA female mice (Mus musculus) were induced to superovulate by intraperitoneal (i.p.) injection of 10 IU pregnant mare’s serum gonadotropin (A036A02, Intervet) followed by an i.p. injection of 10 IU human chorionic gonadotropin (hCG) (A038A01, Intervet) after 48 h. The oocytes obtained from the oviducts were cultured in M2 culture medium (M7167, Sigma-Aldrich) supplemented with 0.1% hyaluronidase (H4272, Sigma-Aldrich) to separate cumulus. We used metaphase II oocytes in the experiments.

The animal experiments were carried out in compliance with the U.K. Animals (Scientific Procedures) Act, 1986 and were approved by the Ethics Committee of the N.N. Semenov Federal Research Center for Chemical Physics Russian Academy of Sciences.

#### 2.1.2 Parthenogenetic activation of mouse oocytes

The oocytes were induced for diploid parthenogenetic development by 6 h incubation in potassium simplex optimized medium (KSOM; MR-121-D, EmbryoMax) supplemented with 5 mM strontium chloride (439665, Sigma-Aldrich), 2 mM EGTA (E3889, Sigma-Aldrich) and 5  μM cytochalasin B (C6762, Sigma-Aldrich) in a ${\mathrm{CO}}_{2}$-incubator with 5% ${\mathrm{CO}}_{2}$ and 37°C, as described by Kishigami and Wakayama.^20^ Then, the oocytes were washed and cultured in KSOM in a ${\mathrm{CO}}_{2}$-incubator. The oocytes were observed after 24 and 72 h of incubation to assess the development and calculate two-cell embryos and blastocysts. Laser-treated and control groups were cultured in four-well dishes (Nunc) in separate wells.

#### 2.1.3 Colchicine treatment

The oocytes were cultured in KSOM supplemented with 10  μg/mL colchicine (F071, PanEco) for 4 h and then washed twice in M2.

#### 2.1.4 Collection and handling of human oocytes

In our experiments, all human oocytes were obtained from a commercial human donor oocyte cryobank of the Medical Center of ART, LLC (Moscow, Russia). The oocytes were donated for commercial and scientific purposes.

Cryopreservation of the oocytes was carried out by vitrification according to a standard protocol received from the manufacturer of the vitrification medium (Kitazato, Japan) at an interval of 37.5 to 38 h after hCG administration. Oocyte thawing was performed using a Kitazato thawing kit and standard thawing protocol provided by the manufacturer. After thawing, the oocytes were cultured in 35 mm Nunc culture dishes in 35  μL preequilibrated G-TL (Vitrolife, Sweden) culture medium coated with light paraffin oil Ovoil (Vitrolife, Sweden) in a Planer BT37 incubator (Origio, United Kindom) with 5% $O_{2}$ and 6% ${\mathrm{CO}}_{2}$.

After 30 min of incubation, the oocytes were transported to the scientific laboratory for laser processing. Transportation was performed in preequilibrated G-TL medium in 0.25 Eppendorf tubes in an atmosphere of 36.8 deg for 80 min.

Laser processing was performed in 2 to 6 h after oocyte thawing. Then, the oocytes were transported back to the clinical laboratory within 60 min for the intracytoplasmic sperm injection (ICSI) procedure and further cultivation.

#### 2.1.5 ICSI of human oocytes

ICSI was performed in 7 to 8 h after thawing using human donor sperm donated for commercial and scientific purposes. The ICSI station on Ametec (TMC, United States) table was installed on an Olympus IX70 (Olympus, Japan) inverted microscope equipped with a Takanome micromanipulator (Narishige, Japan), and a Tokai Hit heating insert (Tokai Hit, Japan). For ICSI, we used ICSI pipette and Holding pipette from Vitrolife (Vitrolife, Sweden) and OVOIL, G-MOPS Plus, ICSI medium and ICSI dish also provided by Vitrolife.

The oocytes were transferred to an EmbryoSlide plus culture dish (Vitrolife, Sweden) with G-TL culture medium covered with Ovoil Heavy by Vitrolife (Vitrolife, Sweden). The dish was prepared 16 h before using. Prolonged cultivation was performed using an EmbryoScope plus incubator. An atmosphere of 5% $O_{2}$ and 6% ${\mathrm{CO}}_{2}$ was used to prepare the dish and to cultivate oocytes, zygotes, and embryos.

### 2.2 Polarization and Confocal Microscopy

#### 2.2.1 Spindle visualization

The spindle was observed according to the Olympus IX3-MLWCDA condenser manual in polarized light with LUCPLFLN60x lens. The same lens was then used for femtosecond laser DNA surgery. To visualize the spindle, the oocytes were incubated in ${\mathrm{CO}}_{2}$ incubator at 37°C for at least 1 h. The microscope stage was heated to maintain the temperature of 37°C. Keeping the oocytes warm was crucial for spindle visualization.

Before enucleation, the oocyte spindle was visualized using polarized microscopy. Depending on the angle of polarization, we observed a light spot on a dark background, or a dark spot on a light background. The laser affected the middle of this area, where the metaphase plate is localized. After laser exposure, the oocytes were stained for DNA and tubulin and examined for the presence of chromosomes and spindle.

#### 2.2.2 Fluorescent staining and confocal microscopy

The oocytes were stained for DNA and tubulin simultaneously in a 50  μL M2 drop supplemented with 5  μg/mL Hoechst 33342 (B2261, Sigma-Aldrich) and microtubule cytoskeleton dye diluted to 1X (BioTracker 488 Green Microtubule Cytoskeleton Dye, Sigma-Aldrich) for 30 min. Then, the oocytes were placed in a clean medium and imaged on a Zeiss LSM 980 confocal microscope (Carl Zeiss Microscopy, Jena, Germany), 20× Plan-Apochromat objective (NA = 0.8). Single-photon excitation of Hoechst was performed with the 405 nm laser wavelength, and Microtubule Cytoskeleton Dye – with a 488 nm laser. The laser power and detector gain were the same for each sample. The distance between Z-stack slices was 0.47  μm.

Image processing was performed using ZEN blue microscopy software. The Hoechst 33342 signal was calculated as follows: we measured the overall intensity in circles with 15  μm diameter, and then summed the intensity of all slices.

### 2.3 Experimental Groups

In the enucleation efficiency experiments we had two experimental groups: “pre-stained oocytes” and “stain-free” oocytes. “Pre-stained” oocytes were labeled with Hoechst 33342 before femtosecond laser action (enucleation). After laser exposure these oocytes were labeled with Hoechst 33342 again, and after that oocytes were studied by confocal microscopy. “Stain-free” oocytes were exposed to the laser radiation without applying Hoechst 33342. Similarly, after the laser action, oocytes of this group were also labeled with Hoechst 33342 and observed with confocal microscopy.

In viability experiments “pre-stained” oocytes and “stain-free” oocytes were divided into two sub-groups depending on exact location of laser application: “enucleation” and “cytoplasm.” In the “enucleation” group, oocytes were exposed by the laser to the metaphase plate. In the “cytoplasm” group, oocytes were exposed at any place of the cytoplasm outside the metaphase plate or spindle. Therefore, we had four experimental groups: “pre-stained enucleation,” “pre-stained cytoplasm,” “stain-free enucleation,” and “stain-free cytoplasm.” “Control” and “spontaneous activation” oocytes were not exposed to the laser radiation.

### 2.4 Experimental Setup and Enucleation

A detailed scheme of oocyte enucleation is described in our previous work,^13^ with the difference that we used a femtosecond laser TETA (Avesta Project). In these experiments, we used the following irradiation parameters for the metaphase plate: λ=1033  nm, υ=100  Hz, pulse energy 32 and 46 nJ measured in the sample plane after the objective lens (for “pre-stained” and “stain-free” oocytes, respectively, and this gives irradiance of 4.7 and $6.7\times 10^{12}\;W/{\mathrm{cm}}^{2}$), pulse duration 270 fs. These fs pulse energies were chosen for being 10% less then vapor-gas bubble formation threshold, which was determined for each experimental group individually (“pre-stained “and “stain-free” oocytes). Laser radiation was focused with 60× objective lens (NA=0.7). Before enucleation, the metaphase plate was visualized using polarized light illumination. The metaphase plate was then exposed to the laser radiation. Exposure of the whole spindle area was achieved by moving the microscope stage through an integrated external manual controller. The procedure was repeated in three different Z planes (plane of observation and ±3  μm from the observation plane) for a complete exposure of the metaphase plate.

## 3 Results

### 3.1 Mouse Oocyte Enucleation: Efficiency and Development

To study the efficiency of the enucleation, we used “stain-free” oocytes and oocytes pre-stained with Hoechst 33342 (the description of the experimental groups is given in Sec. 2.3). The experiments were carried out in two stages: (1) localization of the spindle or metaphase plate and femtosecond laser treatment; (2) observation of treated oocytes. Stage 1 was performed in the main experimental setup; stage 2 was carried out using a confocal microscope Zeiss LSM 980.

**Fig. 2:**
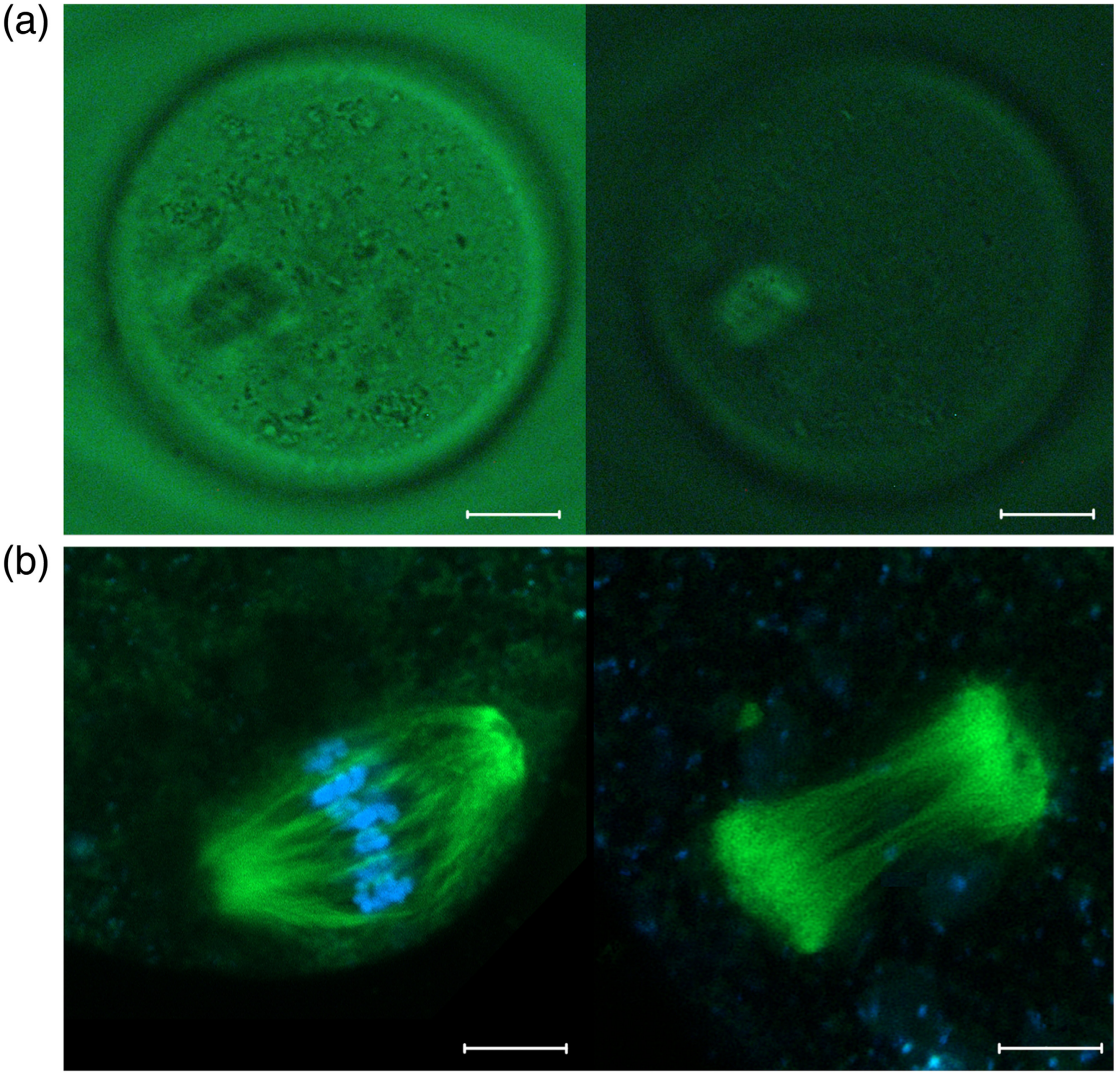
(a) Mouse oocytes imaged by polarized light microscopy. The spindle is visible as a dark spot on a light background light (left) or as a light spot on a dark background (right). Scalebar is 15  μm. (b) Spindle of the intact oocyte (left) and enucleated (without stain) oocyte (right). Microtubules are shown in green, and DNA is shown in blue. Scalebar is 5  μm.

The spindle of the “stain-free” oocytes was detected using polarized light microscopy. Depending on the angle of polarization, we observed a light spot on a dark background or a dark spot on a light background at the spindle localization within oocyte [Fig. 2(a)]. The metaphase plate of “pre-stained” oocytes was detected by UV illumination. After detecting the spindle or metaphase plate, the oocytes were exposed to laser radiation and then examined for the presence of chromosomes and spindle. For this purpose, we used Hoechst dye on “stain-free” oocytes and BioTracker 488 Green Microtubule Cytoskeleton dye on both “stain-free” and “pre-stained” oocytes. Further observations were performed using a confocal fluorescent microscope.

**Fig. 3:**
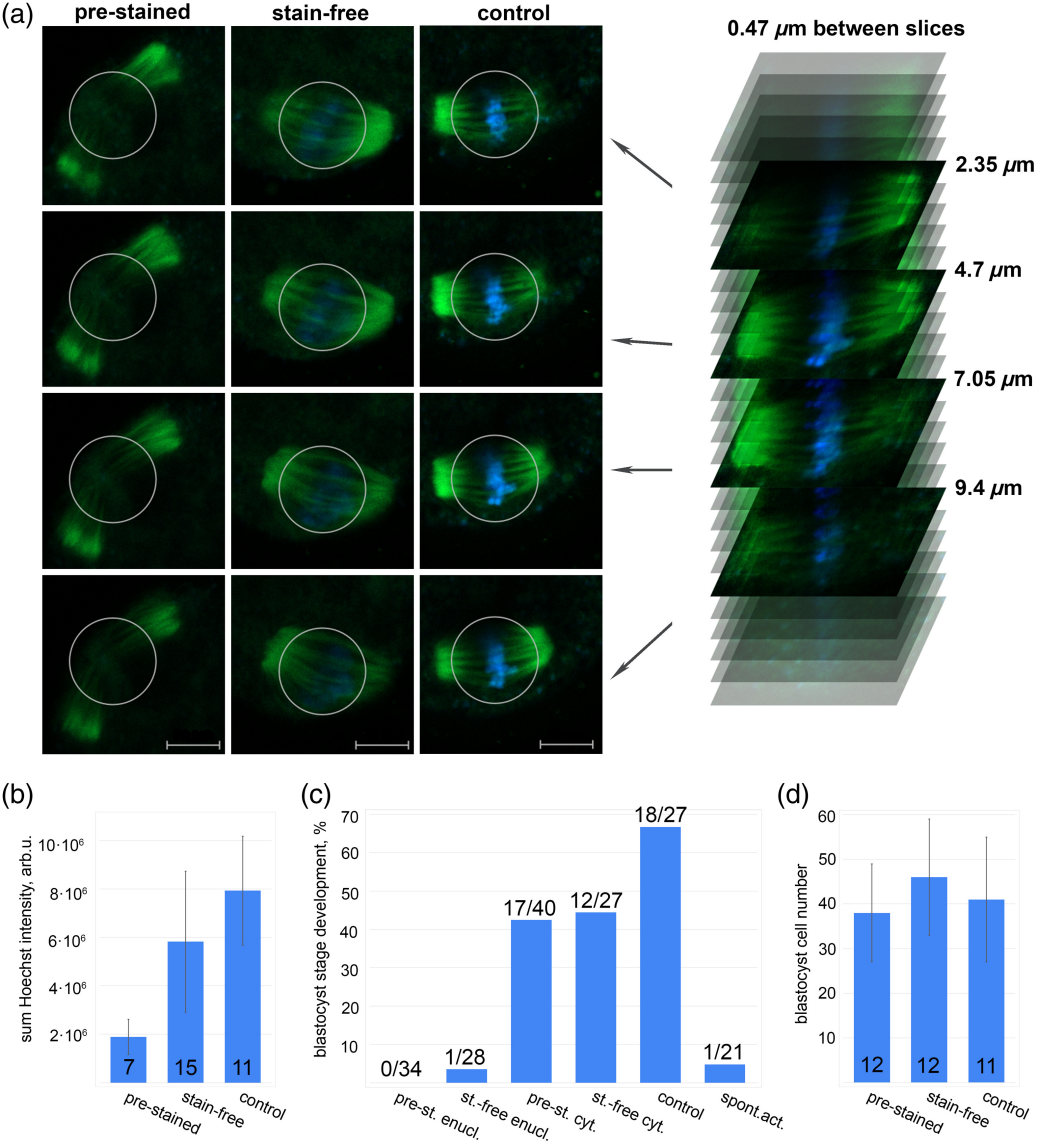
(a) Images of the spindle taken from different depths of a Z-stack. The Hoechst intensity (blue color) was measured within the circle in each slice, its diameter was the same for all oocytes. Scalebar is 10  μm. (b) Average sum intensity of the Hoechst 33342 luminescence in enucleated (“pre-stained” – dye was applied before and after the laser action; “stain-free” – dye was applied only after the laser action) and control oocytes. The number of oocytes is shown within the bars. (c) Parthenogenetic development of oocytes to the blastocyst stage after laser action. The bars show % of blastocyst stage formation for the following groups: “pre-stained enucleation” (pre-st. enucl.), “stain-free enucleation” (st.-free enucl.), “pre-stained cytoplasm” (pre-st. cyt.), “control,” and “spontaneous activation” (spont.act.). The numbers above the bars indicate the exact number of blastocysts/number of irradiated oocytes. (d) The number of cells in blastocysts obtained from oocytes irradiated to the cytoplasm with or without staining. The number of calculated blastocysts is shown within the bars.

In some “stain-free” oocytes the metaphase plate was completely eliminated [Fig. 2(b)], but in others we found DNA luminescence. Thus, to measure the efficiency of femtosecond laser enucleation, we obtained Z-stacks of the spindle area [Fig. 3(a)] and then calculated the total intensity of the Hoechst luminescence along the all Z-stack in “control,” “stain-free,” and “pre-stained” oocytes [Fig. 3(b)]. The total Hoechst intensity was significantly lower in both “stain-free” and “pre-stained” oocytes compared to the control ones (T-test, p<0.00001 and p=0.003 subsequently). However, the average sum Hoechst intensity in “pre-stained” oocytes was nearly two times lower than in “stain-free” oocytes.

To assess viability of the oocytes as well as the efficiency of the enucleation, we performed diploid parthenogenetic activation, which induced development to the blastocyst stage. Both “pre-stained” and “stain-free” enucleated oocytes were unable to form blastocysts [Fig. 3(c)], whereas “stain-free cytoplasm” and “pre-stained cytoplasm” oocytes (irradiated at any place of the cytoplasm outside the metaphase plate) developed similarly to the control oocytes (“control”). The average number of blastocyst cells did not differ significantly between “cytoplasm” and “control” oocytes [Fig. 3(d)].

### 3.2 Human Oocyte Enucleation

**Fig. 4:**
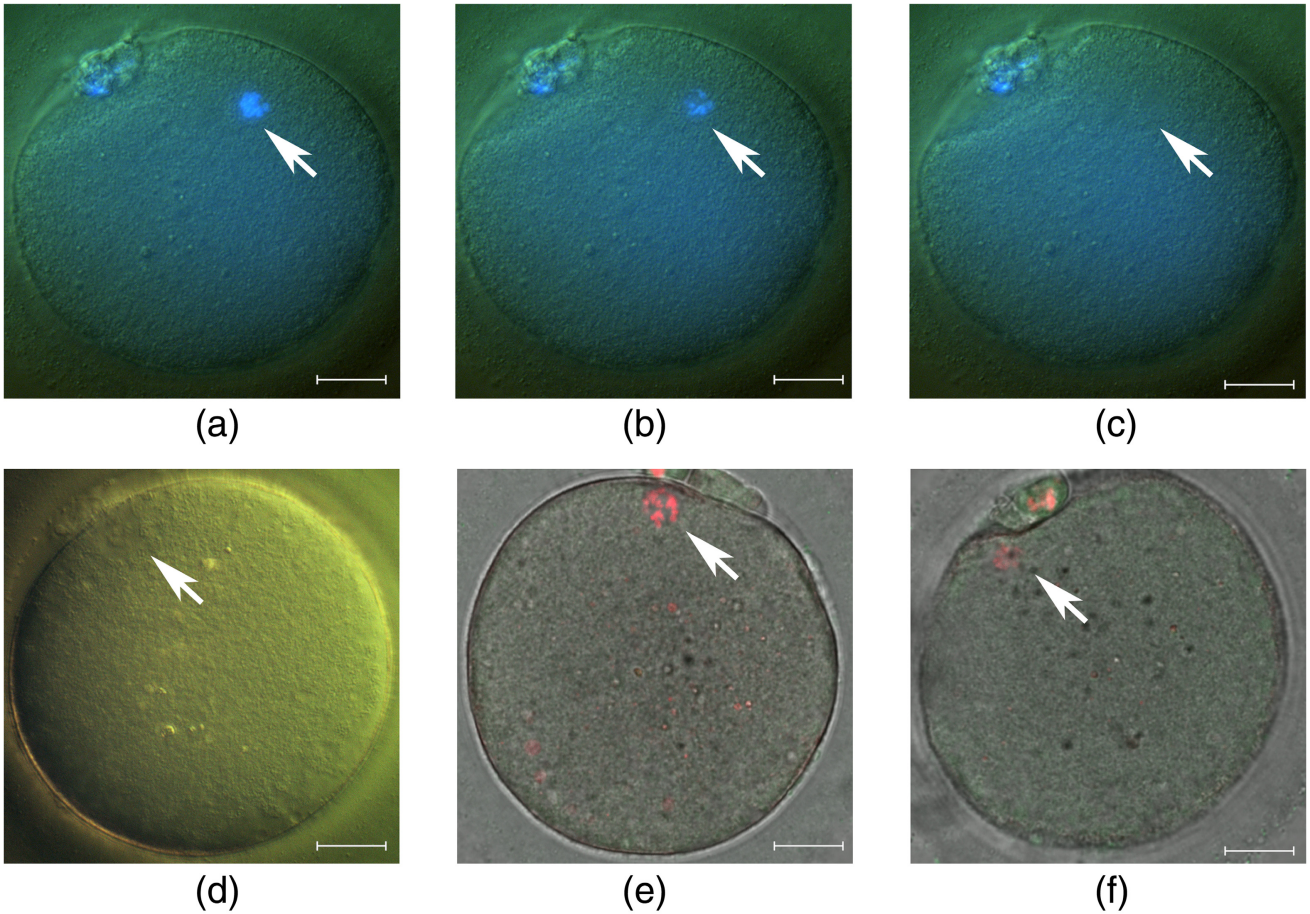
“Pre-stained” (a)–(c) and “stain-free” (d)–(f) human oocyte enucleation. Oocyte (pre-stained with Hoechst 33342) before laser action (a), during (b), and after (c) exposure. (d) Stain-free oocyte visualization by DIC. The opaque area shown by the arrow was irradiated in enucleation experiments. (e) Oocyte with cytoplasm exposed to laser radiation. (f) Oocyte with the metaphase plate exposed to laser radiation. Scalebar is 20  μm.

We also performed “pre-stained” and “stain-free” enucleation of human oocytes. “Pre-stained” human oocytes were illuminated with UV and exposed to laser radiation in the same way as mouse oocytes [Figs. 4(a)–4(c)]. After laser exposure, the oocytes (n=11) were cultured overnight and examined for their viability the following day. We found only one atretic oocyte; another 10 oocytes had normal morphology.

“Stain-free” human oocyte enucleation was complicated by the fact that the oocyte spindle in metaphase II was not visualized by polarization. Therefore, for the enucleation, we irradiated the opaque area near the polar bodies visible by DIC [Fig. 4(d)]. Oocytes of another experimental group (“cytoplasm”) were irradiated beside the metaphase plate (n=7). After exposure, we revealed chromosomes using confocal microscopy [Figs. 4(e) and 4(f)] and found that the total intensity of DNA luminescence in enucleated oocytes was almost 35% lower than in control ones ($40.9\cdot 10^{3}\pm 13.5\cdot 10^{3}$ versus $63.7\cdot 10^{3}\pm 23.2\cdot 10^{3}$, arbitrary unit). All oocytes were subjected to ICSI. In the “cytoplasm” group, five oocytes formed two pronuclei, one oocyte was three-pronuclear, and another one had no pronuclei. Enucleated oocytes (n=4) failed to form two pronuclei; however, two oocytes had one pronucleus each, and another two oocytes had no pronuclei.

### 3.3 Spindle Reorganization

**Fig. 5:**
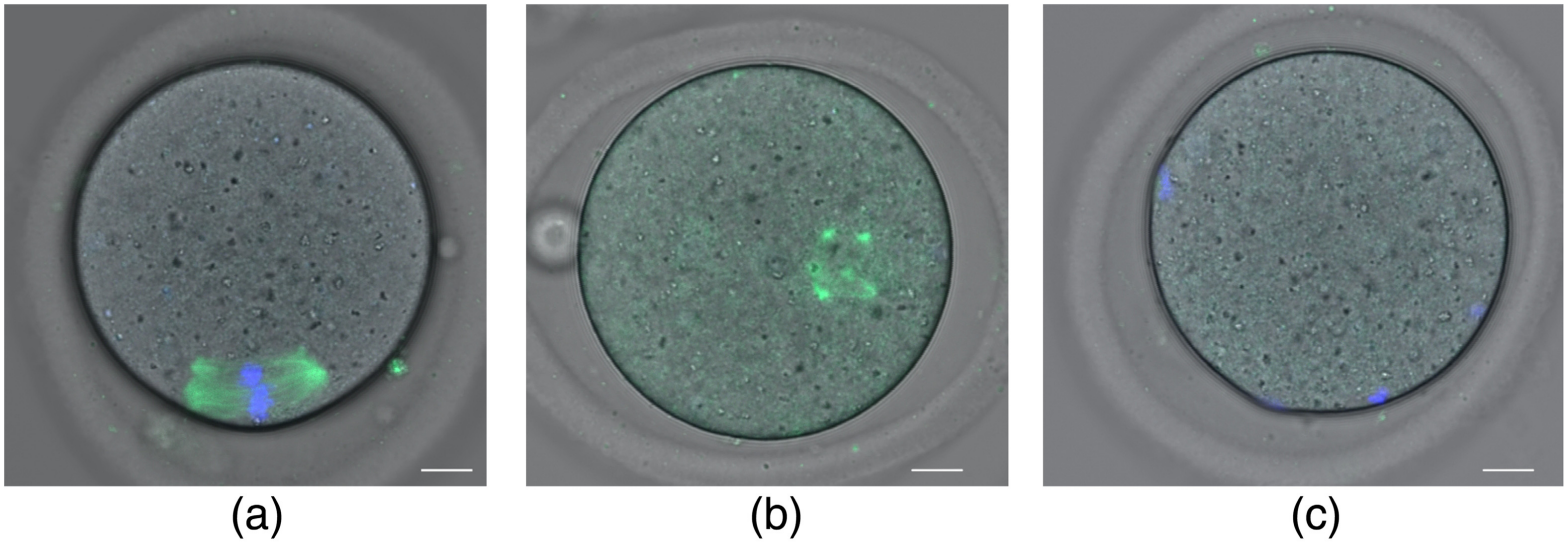
Spindle structure in control, enucleated (without stain), and colchicine-treated oocytes: (a) two poles, (b) three and more clusters of polarization, and (c) depolymerization. Scale bar is 10  μm.

We observed the spindle condition in “stain-free” oocytes after femtosecond laser enucleation. The oocytes were stained for tubulin 1 to 2 h after laser exposure. While control oocytes had primarily a typical bipolar structure of the spindle [Fig. 5(a)], enucleated oocytes lost their normal spindle morphology [Fig. 5(b)]. However, this was not a complete depolymerization specific for tubulin poison, namely colchicine [Fig. 5(c)]. After DNA destruction, the most probable outcome for microtubules was polymerization in several (3 to 7) clusters (Table 1).

**Table 1:** Spindle morphology in control, enucleated (without stain) and colchicine-treated oocytes.

Group	N oocytes	Two poles	Three and more clusters	Depolimerization	Other
Control	22	15 (68%)	2 (9%)	0	5 (23%) – division
Enucleation	23	1 (4%)	14 (61%)	0	4 (17%) – fragmentation and 4 (17%) – division
Colchicine	21	3 (14%)	0	16 (76%)	2 (10%) – division

## 4 Discussion and Conclusion

In this work, we performed an effective and low-invasive preparation of a recipient cytoplast using femtosecond 1033 nm laser radiation with or without applying a fluorescent dye. Laser oocyte enucleation has already been performed in mice^2^ and pigs;^3^ however, laser inactivation of the metaphase plate without a fluorescent dye has not been shown yet. Our study applied polarization microscopy for spindle visualization, and we irradiated the metaphase plate based on the spindle location.

Although pre-stained oocyte enucleation appears low-invasive and effective,^13^ the application of dye is not suitable for clinical use. Therefore, we examined enucleation of the oocytes without using dye. Polarized light microscopy allowed us to reveal the spindle location, which we used as a target for metaphase plate irradiation in mouse oocytes.

The efficiency of stain-free metaphase plate destruction in mouse oocytes varies in a wide range and can achieve almost 100% (Fig. 1). The dye allows for more accurate enucleation as it helps to find the exact location of the metaphase plate and absorb the laser more effectively. Unlike pre-stained oocyte enucleation, enucleation without dye requires higher laser intensities because the dye, acting as a photosensitizer, reduces the breakdown threshold.^14^^,^^21^ For this reason, enucleation of “pre-stained” oocytes was performed with laser pulse energy 32 nJ (irradiance $4.7\times 10^{12}\;W/{\mathrm{cm}}^{2}$), which is 1.43 times lower than energy used in experiments with “stain-free” oocytes. For each experimental group (“pre-stained” and “stain-free” oocytes), we determined the irradiance threshold for vapor-gas bubble formation. The vapor-gas bubble is able to damage the cell, so it was important to avoid bubble formation. Therefore, we used irradiance 10% lower than corresponding threshold irradiance, in order to omit vapor-gas bubble formation.

Compared to dye-mediated DNA destruction, stain-free enucleation proved to be nearly half as effective. Nevertheless, this was enough to stop the development of mouse oocytes. At the same time, laser action itself appears low-toxic and low-invasive since affecting the cytoplasm (not the metaphase plate) does not significantly disturb the development. The probability of blastocyst formation, as well as the number of blastocyst cells does not differ significantly between cytoplasm-irradiated oocytes and the control oocytes [Figs. 3(c) and 3(d)]. The presence of a stain does not affect the possibility of reaching the blastocyst stage, nor the number of its cells.

The spindle was successfully visualized in almost all freshly-recovered MII mouse oocytes. If the spindle was not visible, we did not use the oocyte in the experiment. In the experiments with human oocytes, we had a small number of samples, and therefore were not able to choose the best ones. Unfortunately, we did not observe spindle birefringence in human oocytes under polarized microscopy, thus we applied DIC microscopy to improve visualization of the spindle or metaphase plate. To some extent, this helped us to find an approximate location of the metaphase plate [Fig. 4(d)]. Our preliminary results show the principal possibility of human oocyte enucleation by 1033 nm femtosecond laser with applying a dye and even without a dye. However, further investigation of human oocyte enucleation by laser is required.

The appearance of the human oocyte spindle depends on the egg maturity.^22^ In addition, the spindle is sensitive to many factors, including temperature^23^ and osmolarity.^24^ It was difficult to control all these factors in our experiments because the affiliated medical center and our scientific laboratory are located in different places, and the spindle might have been impaired due to oocyte transfer conditions. Moreover, freezing and thawing also affect the spindle incidence, location and morphology.^25^ However, spindle visualization by polarized light microscopy is applicable in human clinical practice.^22^^,^^26^^,^^27^ Therefore, using laser enucleation in human oocytes is expected to be effective under accurate visualization of the spindle.

Despite the fact that the spindle and the metaphase plate are located very close, the laser destroyed only the metaphase plate (Fig. 1). It should be noted that microneedle aspiration completely eliminates the spindle.^16^ For a short period after enucleation (up to 30 min), the spindle remained intact, but then, within 3 to 4 h, it lost its bipolar structure and reorganized into several small clusters. The laser appears to have destroyed the MTOCs, composed of centromeres and kinetochores, and may have spread undetectable remains of MTOCs throughout the oocyte. Those remains became the attractors of microtubule polymerization.

It is currently unclear what remains of DNA after laser irradiation. The absence of fluorescence under confocal microscope observation only implies the absence of a binding structure for the fluorescent dye, such as DNA double strand in the case of Hoechst dye. There is evidence of single and double strand DNA breaks under femtosecond laser irradiation.^15^ However, the extent of DNA destruction after femtosecond treatment should be considered in subsequent investigations. The potential of left DNA fragments to affect further development (if using enucleated cytoplasts for cloning or other assisted reproductive techniques) is also the question of great interest and importance.

## Data Availability

Data underlying the results presented in this paper are not publicly available at this time but may be obtained from the authors upon reasonable request.
